# Professional Bias in Theory and Practice

**Published:** 1884-09

**Authors:** 


### Editorial.

### PROFESSIONAL BIAS IN THEORY AND PRACTICE.

Mystification in theory and blundering in practice generally spring from erroneous views of facts or misapplication of principles. A vein of perverted reasoning runs through the mental operations of most medical men upon some of the issues which come up for decision in their daily experience as practitioners. There is a misconception of the elements which enter into the groundwork of a diagnosis, or misapprehension of the measures appropriate to the cure, from a preconceived notion of the surroundings of the case. In other words there is a monomonia in the intellectual organization of the majority of those who enter the medical profession, which pervades everything to which their thoughts are directed, and it crops out not only in their individual efforts, but in their co-operative undertakings. In some instances it is manifested in obstinate egotism, but in others by subserviency to the dictum of an arrant impostor—the blind following the blind.

The prevalence at different periods of medical history of views which were exclusive, indicates the preoccupation of the mind with dogmas, to such an extent that at one time debility was the sole indication to be met, and stimulants only were used in the treatment of diseases. Again, excitation had to be met by bloodletting and depressing agencies of every kind, so as to combat supposed inflammatory tendencies. In a later era nervous disturbances predominated throughout the entire catalogue of diseases, and remedies of the antispasmodic order came into requisition. The current which set in the direction of these recognized doctrines could scarcely be opposed by any one who might have independence of thought, as it bore down every thing before it. Yet this did not prove its correctness, and we are convinced now of the egregious mistakes of the past, while many amongst us continue the same kind of absurdities, in adopting the hypotheses of prominent members of the profession without that personal investigation which is requisite.

Illustrations of this proneness to recognize authority without proper examination are afforded in the attitude of many medical men as to the use of anesthetics, the resort to antiseptics, the employment of bloodletting, the opposition to alcoholic stimulants, and opiates as medicines, the purgative treatment of diseases, the water cure, external and internal, and all special measures for correcting the ills that flesh is heir to.

At the same time a partisan spirit is manifested in respect to the theories and principles involved in the experimental developments of the microscope and other processes of investigating the origin of diseases. The germ theory is supported by able advocates of its claims, while it is opposed by others equally entitled to consideration, and many good and true men in the profession maintain the position of masterly inactivity.

There is no doubt but that microcosms accompany certain disorders of the organism, yet they may stand in the same relation to the disease that the maggot does to the superficial sore, and develop because of the favorable conditions for propagation in the impaired vitality of the part.

The partiality for one or another view takes possession of the experimenter in such a manner that he becomes oblivious of all that does not favor his favorite solution, and thus it is in our own country as well as abroad, that we have demonstrations of widely different results according to the standpoint from which experiments are conducted.

The recent passage at arms between Drs. Formad and Shakespeare reminds us forcibly of the duel in the dark, when upon return of the light each antagonist was found snugly in his own corner, without suffering any injury himself or doing any damage to his foe. And the notable parley of Drs. Thomas and Barker only served to show the difference between tweedledum and tweedledee in the lights of their respective reflectors, as no one has been able to discover any radical or fundamental divergence in the pathological doctrines of the contestants. It was the old case of looking at the same thing through glasses of different color. The hygienic spat between Drs. Day and Chaillé belongs to the same order of ill defined antecedents from which to draw conclusions; and hence each taking his own horn of the dilemma. These conspicuous examples are cited simply to impress the main point, that professional bias has too much sway over all classes of medical men in shaping their opinions.

Those who are not caught in the haze of uncertainty and doubt, are apt to be dazed by their preconceived ideas of what ought to appear evident to others, so that there are few who go beyond the beaten path of recorded facts and legitimate induction, who do not fall into a quagmire. They flounder about in the utmost desperation and bespatter all who come in reach of them with the mud and slime by which they are besmeared from head to foot.

It is one of the most ominous signs of the times that some members of the profession who strike out upon their own responsibility, seem only to aim at accomplishing something new, without looking to a trustworthy basis or considering the permanent advantages from such innovations in practice.

To this class belong the aspirants for fame in introducing anesthetics and antiseptics, as it will be noted that Dr. Hofmeir gives two cases of poisoning by vaginal injections of bichloride of mercury in Germany. If it were not after the order of the exclamation when the cow swallowed the grindstone, we might well say "We told you so," in regard to the use of this poisonous article.

If any other evidence of the infatuation of medical men is called for, it is afforded by the wholesale abandonment of the right of private judgment, and entire delivery of themselves to the control and direction of some grand luminary who is supposed to throw a light upon all around. It is well to give honor to whom honor is due; but a beautiful illustration of the distinction to be made between the man and his opinions was recently presented in the gynecological section of the American Medical Association by criticising the views of Dr. S. D. Gross, to whom all felt their indebtedness for much that was valuable in surgical improvements. If the rising men in the different departments of practice should always analyze the propositions coming from even the most distinguished teachers, so as to select that which is useful and reject that which is worthless, it will prove highly conducive to the progress of medical science. Great works and not great names are entitled to our consideration. The records of experience by men unknown to fame are entitled to consideration, in the same manner as the clinical observations of the most distinguished authors; and such facts constitute the statistics which afford a basis for correct practice. Those who have preconceived theories to uphold are more prone to make forced inferences from cases under their treatment, than the general practitioner who seeks only the truth, and if we have the data given correctly, each investigator can make his own deductions.

The recognition of good faith on the part of writers whose names have not become a passport to public favor, might reveal merit in the productions of many zealous laborers among the younger members of the medical profession, and there is nothing so unfavorable to scientific progress as toadyism to great names. Additional value might be given to the selections in quarterly retrospects, periscopes, and epitomes of the advances in all the several departments of professional knowledge, if credit should be given to the journal from which they are taken without affixing the names of the authors. The republication of articles or abstracts without making known the writer's name or location, would allow readers to exercise an unbiased judgment upon their intrinsic worth, instead of pinning their faith to some prominent specialist whose only distinction springs from doing something in some different manner from that generally adopted by the profession.

It should not be understood that the original publication be made without the author's name, but with this guarantee of personal responsibility for the facts stated in opinions advanced, the reproduction of all contributions to medical literature without the names of the writers is calculated to promote a wholesome collection of data by ridding the selections of the domination of those in authority by virtue of their positions or their associations in medical circles.

Far be it from us to detract from the prestige of well-earned prominence of a medical man in his relations with his colleagues; and it is understood that some among us deserve a recognition that cannot be claimed by others. A full measure of appreciation is extended to the worthy follower of Esculapius who fulfills his mission in faith, hope and charity with his associates, and who goes about doing good in the true spirit of peace and good will to all mankind.

But it is urged that fictitious qualities or high-sounding titles do not beget the genuine attributes of greatness, any more than the gaudy plumage of the peacock can impart to the jackdaw the beauty and grace which it covets. And it is simply claimed that every man in the medical ranks shall pass for what he is worth, not overestimating those of high estate, nor underrating such as may hold a more humble position in life. The highway to honorable distinction should be opened up alike to all without let or hindrance from professional bias or prejudice.

